# Cultural Heritage Resource Development and Industrial Transformation Resource Value Assessment Based on BP Neural Network

**DOI:** 10.1155/2022/2288358

**Published:** 2022-09-19

**Authors:** Xinyu Liu, Yujie Li, Zihao Zhang, Qianzheng Wang

**Affiliations:** ^1^School of Performance and Cultural Industries, University of Leeds, Leeds LS2 9JT, Leeds, UK; ^2^College of Science, Xi'an University of Architecture and Technology, Xi'an 710000, Shaanxi, China; ^3^School of Computing, University of Leeds, Leeds LS2 9JT, Leeds, UK; ^4^School of Business, University of Leeds, Leeds LS2 9JT, Leeds, UK

## Abstract

Mining and utilizing cultural heritage resources and creating and developing creative and cultural industries have become the priority direction of economic development, setting off a wave of cultural heritage resource development, and industrial transformation. Which cultural heritage resources can have the high value of industrial transformation has become one of the research topics that have attracted much attention. In view of this problem, research is of great significance to the development of cultural heritage resources and the field of industrial transformation. With the in-depth research on resource development and industrial transformation, the research on artificial neural network (ANN) in cultural industry transformation is gradually carried out, and its performance advantages are of great significance to solve the problem of value evaluation. This paper aims to study the application of the value assessment method based on the BP neural network (BPNN) in the development of cultural heritage resources and industrial transformation. Through the analysis and research of BPNN and cultural heritage resource development and industrial transformation, it can be applied to the construction of resource value assessment methods to solve the problem of improving the value level of cultural heritage resource development and industry. This paper analyzes BPNN, cultural heritage, and value evaluation methods, conducts experimental analysis on the performance of the algorithm, and uses related theoretical formulas to explain. The results show that the evaluation method has passed the random consistency test, and the results are valid. The obtained index popularity weight value is 0.134, the economic benefit weight value is 0.093, and the resource correlation degree weight value is 0.074, which can be used as the key criteria for resource value evaluation. The classification of resources through resource value assessment can provide theoretical support for the development and industrialization of cultural heritage and can meet the needs of improving the value and quality of cultural heritage development and industrial transformation, and the development level and satisfaction have been greatly improved.

## 1. Introduction

Under the upsurge of cultural resources and heritage protection, developers have carried out development and industrial transformation of traditional cultural heritage. In the past, the development of most of the cultural heritage was too blind, and the value generated by the transformation industry was uneven, and it could not meet the increasing demands of people in terms of economic development and artistic aesthetics. Resource value assessment is an evaluation method to assist decision-making to quantify the value of the assessment object, which can solve the problem of comparing the value of different types of resources. Due to its advantages in comparison, it has been applied to various fields to successfully solve various decision-making problems. It quantifies the value of several different types of resources according to the set indicators according to the purpose of evaluation and judges the value of a certain resource by sorting the calculated values to assist decision-making. In the development of cultural heritage, its content exists in various forms, and how to identify the development value of a large number of cultural heritage resources has far-reaching significance for its development and industrial transformation. ANN is an operation model, which is composed of a large number of nodes connected to each other. However, ANN has a better effect on the value evaluation problem to be solved and has less restriction, so its application range is very wide. In recent years, scholars have used ANN for cultural resource evaluation, but the application and research of BPNN in this area are relatively few. Therefore, it is of great significance to apply BPNN in this paper to solve the research on resource value evaluation in the development of cultural heritage resources and industrial transformation.

At present, with the continuous advancement of the development of cultural heritage, more and more scholars have explored the development and industrial transformation of cultural heritage resources. Among them, in order to solve the problems of insufficient resource integration and serious destruction of cultural heritage, Pang et al. used the geographic information system platform to establish a basic database of historical heritage [1]. Rouhani introduced the protection and development of cultural heritage in peacebuilding and economic development and put forward his own views on the path of protection of cultural heritage resources in the future [2]. Yi et al. focused on the latest progress of China's national cultural information resource sharing project to understand people's expectations and requirements for cultural heritage development projects [3]. In order to better develop cultural heritage resources, Heitman et al. studied an extensive and centralized repository of cultural heritage information, providing opportunities for research and facilitating the cocreation of a cultural heritage information exchange community [4]. In order to develop smart cultural tourism services, Ruzic identified and sorted cultural heritage resources that appeared on social media and marked them [5]. However, these are not good for the development of the transformation industry of cultural heritage resources.

BPNN can be used in the value assessment of cultural heritage resources, and it has a good effect on the processing speed of the assessment data and the accuracy of the assessment results. Among them, cultural heritage resources can arouse heated discussions on social platforms. Ding and Tian proposed an algorithm called PRABP to predict the number of retweets in cultural heritage [6]. In order to analyze the tourism volume of cultural tourism resources, Li et al. proposed an effective model based on the Baidu index to predict tourism volume [7]. Zhang et al. proposed a method based on the improved artificial fish swarm algorithm neural network algorithm to improve the prediction accuracy of the audience's preferences for cultural heritage [8]. In order to improve the efficiency of resource value evaluation, Zhang et al. developed a simulator using BPNN to predict the exploitability of cultural heritage resources [9]. These methods improve the efficiency of resource value evaluation to a certain extent, but the accuracy is not high.

In order to solve the problem of the above-mentioned low level of cultural heritage resource development and industrial transformation, this paper uses BPNN to analyze the development of cultural heritage resources and evaluates various indicators to find high-value cultural heritage resources. The innovation of this paper is using BPNN and cultural heritage development and industrial transformation to analyze how BPNN, cultural heritage resources, and value assessment methods play a role in the research on cultural heritage resource development and industrial transformation resource value assessment based on BP neural network. This paper expounds the proposed resource evaluation method. Through the evaluation, this paper finds that this method can improve the accuracy of cultural heritage resource value evaluation and improve the level of industrial transformation.

## 2. Method of Cultural Heritage Resource Development and Industrial Transformation Resource Value Assessment

**Figure 1:**
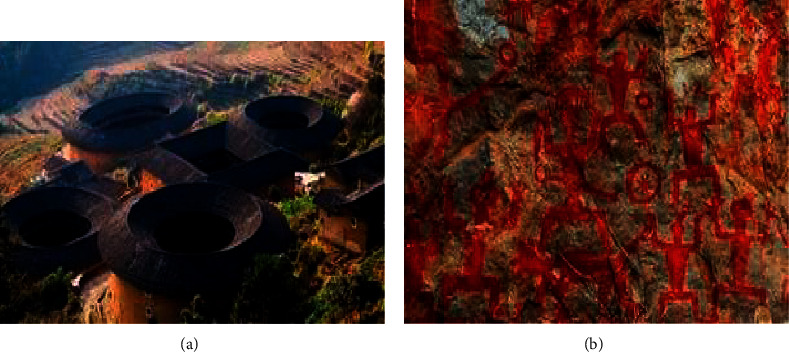
Cultural heritage resources: (a) Fujian Tulou; (b) Huashan Murals.

The 21st century is an era of cultural and economic integration. The cultural industry and modern technology have become the driving force of economic growth, but the quality of the emerging cultural heritage development projects is uneven, and there are many problems, such as the low actual industrial value of the project and resource damage caused by excessive development. Therefore, it is very important to study the development of cultural heritage resources and the evaluation of the value of industrial transformation resources [10]. The cultural heritage resources are shown in Figure 1: Figure 1(a) is the Fujian Tulou, and Figure 1(b) is the mural of Huashan Mountain.

Through the investigation, it is found that the current research on cultural heritage resource development and industrial transformation resource value assessment based on BP neural network is not complete, and most of the research focuses on the theoretical analysis of cultural resource development strategies or phenomena, so this paper proposes a research on the value assessment of cultural heritage resources using BP neural network [11, 12]. This paper analyzes the related methods of BPNN, cultural heritage resource development, and resource value evaluation and proposes an evaluation method for cultural heritage development and industrialization resource value. Through experimental analysis, it is found that cultural resource value assessment can promote the development of cultural heritage and the better development of industrialization.

This paper mainly introduces the research background of cultural heritage resource development and industrial transformation resource value assessment based on the BP neural network, draws out the problems to be solved to illustrate the purpose and significance of this paper, then makes a general analysis of the research status of cultural heritage resources development and industrialization and the application of BPNN, and explains the content and innovation of this paper. At the same time, the organization structure and method of this paper are described, and the related methods of BPNN, cultural heritage resource development, and industrialization and resource value assessment are analyzed and described; then, the data source of this paper is explained in detail; after arranging the data, this paper analyzes the development trend of heritage resource development and industrial transformation, the preference of cultural heritage types, the satisfaction of former cultural heritage development projects, and the results of the comparison of various levels within the evaluation index system and different cultural heritage resource projects and draws conclusions; finally, the full text is summarized.

### 2.1. BPNN Method

BPNN is an inverse operation of error, which optimizes and repairs the weights of each neuron by sending out information until the independent error of each neuron is obtained and the expected result is obtained. The algorithm has two main processes, one is forward propagation of data, and the other is reverse propagation. When the sample data enter the test program, the final result is sent to the output layer by computing each hidden neuron. The error between the result and the expected result cannot be ignored, and the program enters the antimissile step. The process of this process is mainly to amortize the errors of the output layer to the front through hidden neurons to provide data. On the basis of adjustment and modification, obtaining data according to the output result, and run the program for many times to finally obtain the desired output result. Such resource value calculation will take the influence of each factor into account more comprehensively, as the weights between the various factors need to be modified several times to obtain valid procedural results [13].

**Figure 2:**
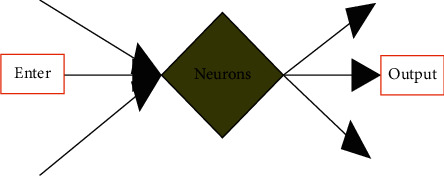
Neuron structure.

The neural network is developed under the inspiration of the neurons of the human brain, and it forms its own processing method by systematically and abstractly processing the data. A neuron is the smallest unit of structure in BPNN [14]. Its structure is shown in Figure 2.

The input of data can be expressed as a stimulus to neurons, and the output of data can be expressed as simple responses of neurons [15]. The relationship between them is shown in the following formulas:

(1) $$\begin{array}{c} R_{j}\overset{n}{\underset{j=1}{\sum }}q_{ij}z_{j}-\alpha_{j}\text{,} \end{array}$$

(2) $$\begin{array}{c} o_{i}=h\left(R_{i}\right)\text{.} \end{array}$$

Among them, *z*_*j*_ is the input variable; *α*_*j*_ is the threshold; *q*_*ij*_ is the weight of adjacent neurons; *h*(*R*_*i*_) is the transfer function.

**Figure 3:**
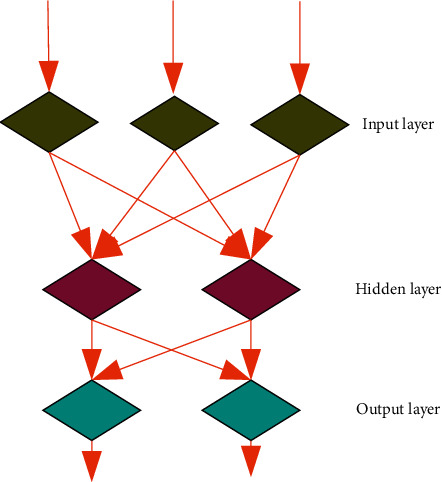
Network structure.

BPNN means a multilayer feed-forward network that only propagates the signal from the back to the front [16]. BPNN supports N-to-N multipoint input and multipoint output mapping with a very high degree of nonlinearity. Its basic structure is shown in Figure 3.

As shown in Figure 3, the neural network training is mainly carried out in the data processing process. Through the forward feedback of the network, the model automatically processes the operation to reduce the error and change the weight between the model layer and each node. Stop training if the threshold feedback errors for the layers and the model's control variables are within the desired accuracy [17, 18]. In practical applications, various training methods can be used to shorten the time of model training, optimize the effect of model training, and further improve the convergence speed of model training. The most commonly used method to improve model training efficiency is gradient descent. It is a first-order optimization algorithm, which iteratively searches for the specified step distance point in the opposite direction of the gradient corresponding to the current point on the function to find the minimum value.

The neurons of BPNN learn general linear functions by default as basic functions. Its expression is shown in the following formula:

(3) $$\begin{array}{c} u=\overset{n}{\underset{j=1}{\sum }}q_{j}z_{j}-\alpha =z^{\prime }q-\alpha \text{.} \end{array}$$

In order to meet the requirements of increasing partial differential equations and monotonicity for specific intervals, the Sigmund function can be used as the output function of the BPNN. The expression is shown in the following formula:

(4) $$\begin{array}{c} h\left(z\right)={\left(1+e^{-\beta z}\right)}^{-1}\text{.} \end{array}$$

Then, the derivative function expression is obtained as shown in the following formula:

(5) $$\begin{array}{c} h^{\prime }\left(x\right)=h\left(z\right)\left(1-h\left(z\right)\right)\text{.} \end{array}$$

The forward propagation of data information is analyzed as follows.

The data input from the first-layer network will be processed by the first-layer and second-layer network connection functions, and the process is shown in the following formulas:

(6) $$\begin{array}{c} nt_{j}=\overset{n}{\underset{i=0}{\sum }}r_{ij}z_{i}\left(j=1,2\cdots m\right)\text{,} \end{array}$$

(7) $$\begin{array}{c} u_{j}=h\left(nt_{j}\right)\left(j=1,2\cdots m\right)\text{.} \end{array}$$

The processed information is then output through the connectivity between the second- and third-layer networks to complete the simple forward propagation of data information. The process is shown in the following formulas:

(8) $$\begin{array}{c} nt_{k}=\overset{m}{\underset{j=0}{\sum }}r_{ik}u_{j}\left(k=1,2\cdots l\right)\text{,} \end{array}$$

(9) $$\begin{array}{c} o_{k}=h\left(nt_{k}\right)\left(k=1,2\cdots l\right)\text{.} \end{array}$$

The error back-propagation processing of data information is analyzed as follows.

Assuming the sample is obtained as shown in the following formula:

(10) $$\begin{array}{c} Y^{d}=\frac{1}{2}\overset{L}{\underset{K=1}{\sum }}{\left(a_{k}^{d}-o_{k}^{d}\right)}^{2}\text{.} \end{array}$$

Substituting it into the calculation formula of the second to third layers to obtain the following formula:

(11) $$\begin{array}{c} Y=\frac{1}{2}\overset{L}{\underset{K=1}{\sum }}{\left(a_{k}-h\left(\overset{m}{\underset{j=0}{\sum }}q_{jk}u_{j}\right)\right)}^{2}\text{.} \end{array}$$

Among them, *q*_*ij*_ is the weight of each layer, that is, the error, which can be changed by adjusting the weight.

In general, it is hoped that the weight value should be proportional to the negative gradient of the error, and this is due to the continuous reduction of the error in the learning process, so it is obtained as shown in the following formulas:

(12) $$\begin{array}{c} \Delta q_{jk}=-\delta \frac{\varepsilon Y}{\varepsilon q_{jk}}\left(j=0,1,2,\cdots ,m;k=1,2,\cdots ,l\right)\text{,} \end{array}$$

(13) $$\begin{array}{c} \Delta r_{ij}=-\delta \frac{\varepsilon Y}{\varepsilon r_{ij}}\left(i=0,1,2,\cdots ,n;j=1,2,\cdots ,m\right)\text{.} \end{array}$$

Among them, *δ* is the learning rate.

Therefore, for the output layer, the formula (14) is obtained as

(14) $$\begin{array}{c} \Delta q_{jk}=\delta \varphi_{jk}u_{j}\text{.} \end{array}$$

Among them, *φ*_*jk*_ is the error signal. The error signal is shown in the following formula:

(15) $$\begin{array}{c} \varphi_{jk}=\left(a_{k}-o_{k}\right)o_{k}\left(1-o_{k}\right)\text{.} \end{array}$$

Then, the hidden layer is obtained as shown in the following formula:

(16) $$\begin{array}{c} \Delta r_{ij}=\delta \overset{l}{\underset{k=1}{\sum }}\varphi_{jk}q_{jk}u_{j}\left(1-u_{j}\right)Z_{i}=\delta \varphi_{jk}z_{i}\text{.} \end{array}$$

The error signal is then corrected as shown in the following formula:

(17) $$\begin{array}{c} \varphi_{ij}=\overset{b}{\underset{k=1}{\sum }}\varphi_{jk}q_{jk}u_{j}\left(1-u_{j}\right)\text{.} \end{array}$$

Through the above formula, the error adjustment of the second and third layers of the sample can be calculated as shown in the following formulas:

(18) $$\begin{array}{c} q_{jk}\left(b+1\right)=q_{jk}\left(b\right)+\delta \overset{D}{\underset{d=1}{\sum }}\varphi_{ij}u_{j}\text{,} \end{array}$$

(19) $$\begin{array}{c} r_{ij}\left(b+1\right)=r_{jk}\left(b\right)+\emptyset \overset{D}{\underset{d=1}{\sum }}\varphi_{ij}u_{j}\text{.} \end{array}$$

Since the construction of the BPNN model, many fields have been using it for different degrees of research and development, and the applicability of BPNN in resource value evaluation has been enhanced. When using BPNN to deal with complex resource information, regardless of economic, legal, or technical aspects, no matter how large the dependence between the data is, it can make the model predicted value closer to the function of the real value. So, this paper applies it to the value assessment of cultural heritage resources.

### 2.2. Cultural Heritage Development and Industrialization Resource Value Assessment

**Figure 4:**
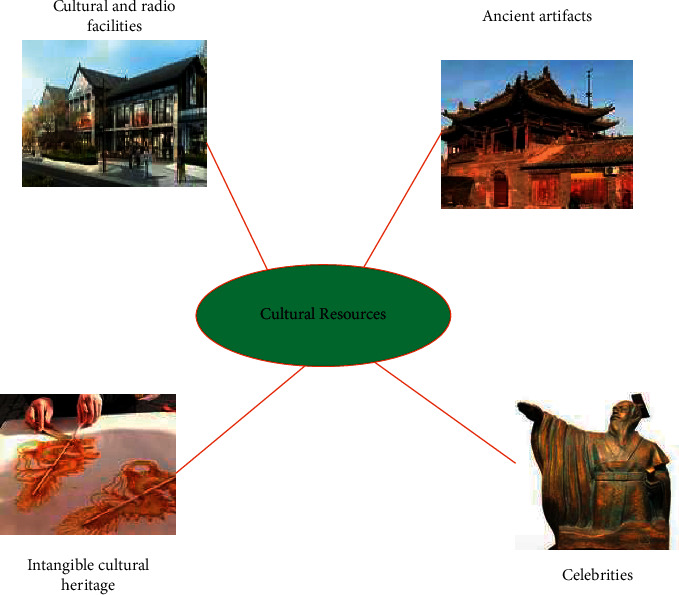
Types of cultural resources.

Cultural resources are the first link in the evolution of cultural forms in the production process of cultural industries and are the basis for creativity. Therefore, evaluating the value of local cultural resources is the first step in the development of local cultural industries. Based on the inherent spiritual and material dual attributes of cultural resources, evaluating and measuring their value has a dual meaning. There are many kinds of cultural resources, and their classification is shown in Figure 4.

As shown in Figure 4, the correct classification of cultural resources is an important prerequisite for the development of cultural resources statistics, and research and cultural industry development strategies. According to the definition of cultural resources, avoid using too empty concepts such as material cultural resources, spiritual cultural resources, and intangible cultural resources.

To evaluate the value of cultural resources, we must first clarify the principles of evaluation. Evaluation should strictly adhere to the principle of objectivity, avoid subjective preference guessing, and adopt one-sided methods when evaluating the value of cultural resources and should combine the mainstream value orientation of the historical development of the cultural industry and use scientific methods. Cultural resources should be measured from a realistic perspective, so as to reflect the real value of cultural resources; evaluation should adhere to local principles, and an important task of cultural resource evaluation is to investigate the heritability of dominant factors in regional cultural contexts. Therefore, it is necessary to pay attention to the discovery of core value resources and the thorough discovery of value when evaluating; the evaluation should be based on qualitative and quantitative principles, cultural resource evaluation is based on the weighted design problem commonly used in systems engineering technology. “Analytic Hierarchy Process” can solve multipurpose system evaluation and decision-making problems caused by multipurpose, inclusive, uncertain, complex, and other characteristics. The overall principle should be followed in the assessment. Cultural resources share their own unique cultural ecological environment. In the industrialization and development of cultural resources, the influence of these cultural and ecological factors needs to be considered. Therefore, assessing the value of cultural resources is not a simple scoring of individual resources but requires looking at it from a holistic point of view.

For the method of resource value evaluation, the first step is to determine the evaluation index of cultural resources and form a set of intuitive evaluation system of cultural resources. Combined with BPNN data analysis, a set of “cultural resources value evaluation index system” is established, and finally, a basic evaluation model is established through the analytic hierarchy process, and this method is used to count and evaluate cultural resources. Through BPNN, the indicators of each layer of the evaluation model are compared and analyzed, and the weight of each indicator is obtained. The weight comparison adopts a top-down approach, determining the weight of upper-level indicators according to the characteristics of cultural resources in industrial development, comparing the weights of lower-level indicators, and refining the top-level indicators to obtain the bottom-level indicators and overall importance. After weighting the indicators of the evaluation model, comprehensively evaluate and analyze the cultural resources of the objects selected in the above-mentioned cultural resources statistics.

## 3. Data Sources for Cultural Heritage Resource Development and Industrial Transformation Resource Value Assessment

This paper conducts an online questionnaire survey on the types of cultural heritage favored by netizens and their satisfaction with the current industrial transformation, and a total of 348 valid questionnaires are obtained. The specific data results of the survey content are shown in Table 1.

Among them, the content information contained in the questionnaire includes the respondents' gender, age, favorite type of cultural heritage, and their satisfaction with the current cultural heritage resource development project. For the satisfaction evaluation part of the questionnaire, 4 options were set, namely very dissatisfied, general, relatively satisfied, and very satisfied, corresponding to numbers 1, 2, 3, and 4, respectively. According to these data, the needs of cultural heritage resource development and industrial transformation resource value assessment can be analyzed.

At the same time, by taking 10 different types of cultural resource development projects as examples, this paper evaluates the resource value of them and classifies them according to the score of the evaluation value. The cultural resource development project data used for the experiment were obtained through Internet search, and the detailed information data are shown in Table 2.

The content of the table includes the label, project name, province, and time of cultural resource development projects used for value assessment.

## 4. Results and Discussion of Cultural Heritage Resource Development and Industrial Transformation Resource Value Assessment

### 4.1. Resource Value Assessment Needs

**Figure 5:**
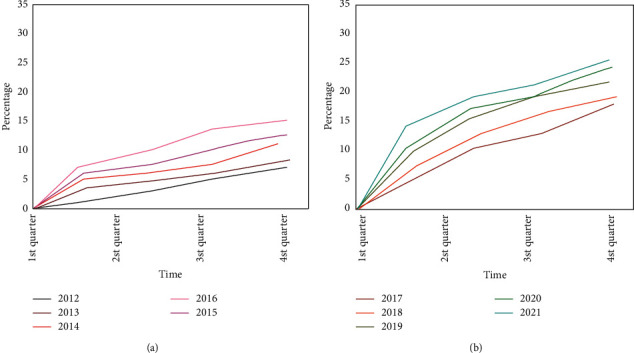
Development trend of cultural heritage resource development projects from 2012 to 2021. (a) 2012–2016, (b) 2017–2021.

This paper analyzes the development trend of cultural heritage resource development and industrial transformation from 2012 to 2021 through the data of cultural resource development projects collected by data mining; and through the distribution of online questionnaires, this paper collects the satisfaction evaluation of netizens according to the current development of cultural heritage resources and industrial transformation. After sorting and analyzing the data, the results are obtained. The development trend of cultural heritage resource development and transformation is shown in Figure 5.

Figure 5 shows the proportion of cultural heritage resource development projects from 2012 to 2021. It can be seen that the overall trend is increasing year by year.  Figure 5(a) shows that the overall number of projects in the development of cultural heritage resources increased from 2011 to 2016, but the growth rate was slow; Figure 5(b) sees an explosive growth in the number of projects for cultural heritage resource development from 2017 to 2021, with a faster growth rate. This is because people gradually realize the value of the development of cultural resources and heritage and advocate their development and industrialization, setting off a wave of upsurge. It can be seen that in the huge number of cultural heritage development projects, the identification of its development value is particularly important for the effective use of limited resources.

**Figure 6:**
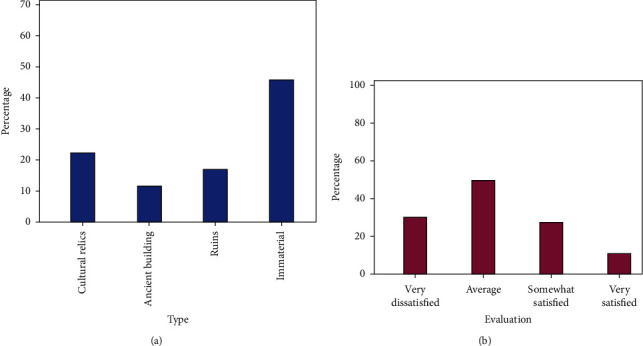
Questionnaire survey results. (a) Type preference distribution. (b) Satisfaction survey.

Through the questionnaire survey, netizens were collected about their preferences for cultural heritage types and their satisfaction with the current cultural heritage development projects. The specific results after data collection and arrangement are shown in Figure 6.

Figure 6 shows the results of an online questionnaire survey on the types of cultural heritage favored by netizens and their satisfaction with the current industrial transformation. From Figure 6(a), we can see the distribution of netizens' preferences for the type of resources. Netizens who like cultural relics accounted for 24.4%, those who like ancient buildings accounted for 11.4%, and those who liked sites accounted for 16.4%, and netizens who liked nonmaterial types accounted for 47.8%. It can be seen that the intangible cultural heritage resources are the most popular among the many types of cultural heritage resource development.  Figure 6(b) shows the degree of satisfaction of netizens with the current cultural resource development project, among which 23.4% of netizens said they were very dissatisfied, 44.6% said they were average, 21.4% said they were relatively satisfied, and 10.6% said they were very satisfied, the proportion of netizens who expressed dissatisfaction reached 68%. It can be seen that the quality of the current numerous cultural resource development projects is uneven, and netizens are generally not satisfied with them. Therefore, it is necessary to evaluate the value of the cultural resources to be developed before the project development to improve the quality of the project and improve satisfaction.

### 4.2. Comparison of Various Levels within the Evaluation Index System

**Figure 7:**
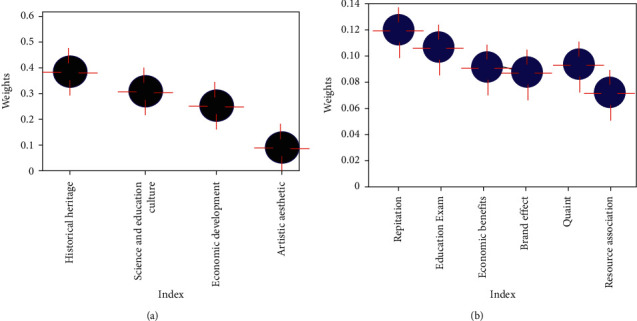
Indicator weights at each level. (a) Criterion layer. (b) Project layer.

This paper calculates the weights of indicators at various levels of cultural resource value evaluation, organizes and analyzes the data, and finally obtains the results shown in Figure 7.

Figure 7 shows the weights of different levels of indicators for the evaluation of cultural resource development value.  Figure 7(a) shows that in the criterion layer, the weight of historical heritage value is 0.35, the weight of economic development value is 0.3, the weight of scientific, educational, and cultural value is 0.25, and the weight of artistic aesthetic value is 0.1. It can be seen that the historical heritage value of the resource has the greatest impact on the evaluation score, and the artistic aesthetic value has the least impact on the result.  Figure 7(b) shows the top 6 weight indicators that have a great influence in the project layer, among which the popularity is 0.134, the education and scientific examination is 0.116, the economic benefit is 0.093, the brand effect is 0.083, the ancient degree is 0.098, and the resource correlation degree is 0.074. It can be seen that focusing on the above six indicators in the development and industrialization of cultural resources can improve the actual quality of the project.

### 4.3. Comparison of Different Cultural Heritage Resource Projects

**Figure 8:**
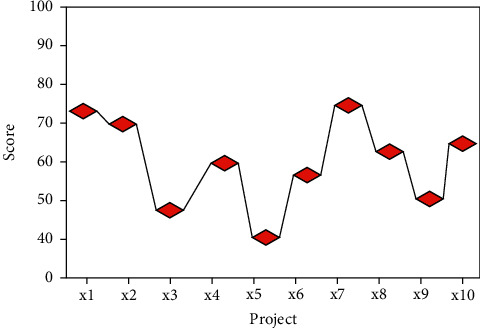
Value assessment results.

This paper evaluates the industrialization development value of a total of 10 cultural heritage resource development projects from X1–X10, and the total evaluation results are shown in Figure 8.

As shown in Figure 8, on the whole, it can be seen that the value evaluation results of different types of cultural heritage resource development projects are still quite different. Most of the items were scored between 50 and 70 points, with the highest overall score for the assessment of item X7 being 73.2 points and the lowest overall score for the assessment of item X5 being 42.3 points.

According to the results of evaluation and scoring, they can be divided into three categories: semidevelopment category (1), pending development category (2), and key protection category (3), as shown in Table 3.

Category 1 in Table 3 is a semideveloped category, and the score range for this category is >70 points. This category is a cultural resource project that is in the development stage and has already achieved certain results. It includes Huangzhong embroidery project, Wudu Mountain Opera project, and traditional incense technology, which are high-industrial value projects. Category 2 is the category to be developed, and the score range for this category is 50–70 points. This category is a cultural resource project that can be developed but has not been industrialized for some reasons, or has just started. It includes the Tibetan Caisha Mandala Painting Project, the Ewenki Clothing Project, the New Fourth Army Shu Four Detachment Lightning Protection Project Site Project, the Shaanxi Jiaxian Ancient Jujube Garden Project, and the Gansu Gaolan Shilan Ancient Pear Garden Project, which are projects of medium industrialization value. Category 3 is the protection category, and the score range for this category is <50 points. Compared with the first two types, this category is a cultural resource with lower economic benefits and more serious damage. It includes Emei Wushu Project and Wannian County Rice Farming Custom Project, which are projects of low industrial value but in urgent need of protection.

## 5. Conclusions

Cultural heritage resources are the treasures of human material and spiritual civilization, and people's requirements for cultural resource development projects are getting higher and higher. The development of cultural heritage resources industrialization is inseparable from the contribution of BPNN. BPNN has been applied in the value evaluation of cultural resources industry development because of its powerful data processing advantages. It can be seen from the comprehensive experimental test that the accurate evaluation of resource value can better promote and improve the quality level of cultural resource industrialization. Through the analysis of the needs of cultural resource value evaluation, it is found that due to the continuous development of the cultural economy industry, a large number of industrialization projects for the development of cultural resources have emerged, so it is particularly important to screen the resource value of these projects. At the same time, in the face of a large number of projects, the quality is uneven. This paper investigates the satisfaction of netizens with the project through a questionnaire survey. Netizens are not satisfied with the current cultural resource development, 68% of netizens expressed dissatisfaction. It can be seen that evaluating the cultural resource value of development projects to improve project quality is also very important to improve satisfaction. By comparing the internal levels of the evaluation index system, it is found that the weight of the influence index on the value of the popularity is 0.134, the education and scientific examination is 0.116, the economic benefit is 0.093, the brand effect is 0.083, the ancient degree is 0.098, and the resource correlation degree is 0.074, and these 6 indicators will be the focus of evaluating the value of resources. By comparing different cultural heritage resource projects, 10 different heritage resource projects are divided into 3 categories according to their scores. Among them, Huangzhong embroidery project, Wudu Mountain Opera project, and traditional incense technology belong to the development category, with a score range of >70 minute. This type of project has a high degree of resource relationship, significant cultural characteristics, strong economic benefits, and high mass participation, and it is relatively easy to form a brand, and the feasibility of industrialization is very high. Tibet Caisha Mandala Painting Project, Ewenki Clothing Project, New Fourth Army Shu Four Detachment Lightning Protection Project Site Project, Shaanxi Jiaxian Ancient Jujube Garden Project, and Gansu Gaolan Shilan Ancient Pear Garden Project belong to the category to be developed, with a score range of 50–70 minute. This type of project has the same advantages as the previous type of project but needs to be properly developed on the basis of better protection and inheritance. Emei Wushu Project and Wannian County Rice Farming Custom Project belong to the protection category, with a score range of <50 points. Due to the special nature of low economic benefits or due to the large degree of damage suffered by such projects, rescue and protection are more necessary than industrial development. It can be seen that the evaluation of the development value of the cultural heritage resources industry provides a basis for guiding the industrialization development and can effectively promote the development of cultural resources. In future research, the focus of follow-up research will focus on how to adapt to the regional conditions of industrial development, what kind of heritage protection mechanism to adopt, how to balance protection and development, and the impact of industrial development.

## Figures and Tables

**Table 1:** Partial data of the content of the questionnaire.

Project	Netizen 1	Netizen 2	Netizen 3
Gender	Male	Female	Male
Age	19	41	27
Type of preference	Cultural relics	Ruins	Immaterial
Satisfaction level	1	3	2

**Table 2:** Ten different types of cultural resource development projects.

Serial number	Project name	Province	Time
X1	Huangzhong embroidery	Qinghai	2008.6.7
X2	Wudu mountain opera	Gansu	2012.8.7
X3	Emei martial arts	Sichuan	2009.4.12
X4	Painting of Tibetan colored sand mandala	Tibet	2013.2.4
X5	Rice cultivation customs in Wannian County	Jiangxi	2020.4.6
X6	Gansu gaolan shilan ancient pear garden	Gansu	2016.3.14
X7	Traditional incense-making techniques	Guangdong	2019.6.7
X8	Evenki clothing	Inner Mongolia	2014.3.6
X9	Lightning protection project of the fourth detachment of the new fourth army in Shu	Shandong	2020.8.6
X10	Ancient Jujube Garden in Jiaxian County, Shaanxi Province	Shaanxi	2018.5.14

**Table 3:** Classification of the value assessment of the industrialization development of cultural heritage resource projects.

Serial number	Project name	Category
X1	Huangzhong embroidery	1
X2	Wudu Mountain Opera	1
X3	Emei martial arts	3
X4	Painting of Tibetan colored sand mandala	2
X5	Rice cultivation customs in Wannian County	3
X6	Gansu Gaolan Shilan Ancient Pear Garden	2
X7	Traditional incense making techniques	1
X8	Evenki clothing	2
X9	Lightning protection project of the fourth detachment of the new fourth army in Shu	2
X10	Ancient Jujube Garden in Jiaxian County, Shaanxi Province	2

## Data Availability

The data of this paper can be obtained upon request.
